# A retrospective study analysing outcomes of the coronectomy procedure at a university dental teaching clinic in Norway

**DOI:** 10.2340/aos.v84.43759

**Published:** 2025-05-26

**Authors:** Umar Sharif, Stein Atle Lie, Cecilie Gudveig Gjerde

**Affiliations:** Faculty of Medicine, Center of Translational Oral Research (TOR), Department of Clinical Dentistry, University of Bergen, Bergen, Norway

**Keywords:** Coronectomy, wisdom tooth resection, inferior alveolar nerve, 3M, partial extraction of 3^rd^ molars

## Abstract

Mandibular third molars (3M) are often associated with pathologies necessitating their surgical removal. Removal of 3M can cause injury to the inferior alveolar nerve (IAN). In coronectomy, a surgical option for 3M that present a significant risk of injury to the IAN, only the crown is removed, and the root/roots are left *in situ*, and may move after surgery.

**Methodology:**

A retrospective review was done to analyse the outcomes of coronectomy procedures performed at the Clinic of Oral and Maxillofacial Surgery, Institute of Clinical Odontology (IKO), University of Bergen (UiB) between 2014 and 2020. During this period, a total of 63 coronectomy procedures were performed. All 63 patients were contacted after approval from the regional ethical committee, and a total of 44 patients consented to participate in the study. Radiographic analysis was done based on panoramic radiographs (OPG), and patient records were analysed to assess which demographic and local factors were associated with root migration along with the number of post-operative complications.

**Results:**

Root migration was not associated with gender, root anatomy, or pattern of tooth impaction. Root migration was observed significantly more often in the younger age group, that is, ≤25 years. There were four reported cases of post-operative complications; two of those cases resolved with systemic antibiotics, while surgical intervention was necessary in the other two cases to remove the remaining roots. There were no reported cases of transient or permanent neurosensory disturbances.

**Conclusion:**

Coronectomy is a viable technique to avoid nerve injury with 3M removal.

## Introduction

Mandibular third molars (3M), or wisdom teeth, are the most frequently retained teeth and are usually associated with multiple pathologies, ranging from recurrent infection to large cystic lesions necessitating the removal of the tooth [1]. These usually erupt between the ages of 18–24 years if the eruption circumstances are favourable. Approximately 40% fail to erupt functionally and become impacted partially or entirely in bone [2].

Impaction of 3M has become more frequent over time, and anthropologists suggest that it is due to the evolutionary reduction of human jaw size resulting in reduced space for wisdom teeth [3]. Surgical extraction of 3M is one of the most frequently performed procedures in oral surgery. One study suggests that 3M surgery accounts for 35.9 to 58.7% of all the procedures performed in oral surgery departments [4].

Indications for removal of 3M vary depending on the respective country’s guidelines, for example, National guidelines from the United Kingdom (UK) and Sweden are based on therapeutic grounds [5]. Norwegian guidelines are based on the recommendations of the Norwegian Center for Evaluation of Medical Methods (SMM). These guidelines favour prophylactic removal of 3M under the age of 30 if removal carries a minimal risk of complications and a predictable risk of pathology development in the future if the tooth is left untreated [6].

Common complications after 3M surgery are usually self-limiting in nature. Significant complications related to extraction of 3M are rare; these include neurosensory disturbances, jaw fracture, and post-operative life-threatening infections. Since the roots of 3M often lie in close proximity to the inferior alveolar nerve (IAN); when this is the case there is a considerable risk that the surgical extraction of roots can cause alteration of the neurosensory innervation by damaging the neurovascular bundle [7]. The incidence of neurovascular injury following mandibular 3M extractions varies, and lies between 1 and 5%, while the rate of permanent dysesthesia is around 0.9% [8]. Earlier studies have shown that IAN and lingual nerve (LN) damage occur with a range from 0.4 to 8.4% for IAN and none to 23.0% for LN [9, 10]. Injury to the buccal nerve (BN) is relatively low compared to the other two branches of the mandibular nerve [11]. Most trigeminal nerve injuries heal spontaneously, and are slightly influenced by age but not by gender [12].

To reduce the risk of nerve injury during 3M removal, different strategies have been discussed in the literature, one of them being partial surgical resection of the tooth called ‘coronectomy’. In this procedure, the coronal part of the tooth is removed, leaving the roots intact and immobilised. This technique was first introduced in the literature in 1984 [13]. Compared to the odontectomy that is , conventional surgical tooth extraction, coronectomy carries 10 times less risk of damage to the IAN [14]. There are no significant differences in post-operative complications, that is, infections and pain, and the incidence of alveolar osteitis is reduced in coronectomy [15]. Root migration is considered a consequence of the eruption forces of the tooth when the coronal resistance is removed, rather than a complication of the surgery [16], and it is the most common post-operative radiological finding after coronectomy. In most cases, this migration is harmless because migration happens in the superior direction away from the nerve, thereby reducing the risk of nerve damage in case of a second surgery. Secondary procedures are seldom necessary but indispensable if the roots come in contact with the oral cavity due to root migration.

Indications for coronectomy include impacted 3M in close proximity to the IAN, with high risk of nerve injury if odontectomy is performed. Several radiographical signs indicate a close proximity of neurosensory bundle to the roots, thereby predicting the risks of damage to the nerve as described by Rood and Shehab [17]. The following radiological signs indicate a higher risk of nerve injury during extraction:

1. darkening of mandibular 3M roots while passing close to the nerve,
2. signs of narrowing, diversion, or deflection of IAN,
3. darkened roots with bifid root anatomy,
4. interruption and loss of margins (white lines) of the nerve canal.

Multiple studies suggest that if two or more of these radiological signs are present, coronectomy should be considered to reduce the risk of damage to the nerve canal [18, 19].

Contraindications include severe infections directly related to the mandibular 3M such as

1. deep caries or periapical pathologies,
2. pulpal pathologies,
3. external root resorption,
4. large cystic lesions related to mandibular 3M.

It has been suggested that this procedure be avoided in the case of medically compromised patients, reduced immunity, or history of radiotherapy to the affected area [8].

This retrospective study is aimed at analysing the outcomes of coronectomy, the use of antibiotics, and associated post-operative complications.

## Material and methods

The regional committee for medical and research ethics (REK – Vest) granted the ethical approval for this study (approval number 460299). The authors reviewed the electronic patient journals (EPJ) and the panoramic radiographs (OPG) at the Department of Oral and Maxillofacial Surgery, Institute of Clinical Odontology (IKO), University of Bergen (UiB), Norway, and traced the patients who underwent coronectomy during the period 2014–2020. These patients were contacted by phone and detailed written information about the project was sent via email or by post. All the participants who agreed to participate in the study signed and returned the written consent forms. The participants in the study were anonymised, and only two final year dental students and the authors had access to the collected data. Participants could withdraw their consent at any given time without any explanation. No economic incentives were offered to the participants.

### Study design

The study was designed as a retrospective study. Indications for surgical treatment of 3M were based on the National guidelines from the Norwegian Center for Evaluation of Medical Methods [6]. Indications for coronectomy were presence of two or more risk factors according to the above-mentioned criteria of Rood and Shehab [17].

To be included in the study sample, four inclusion criteria were devised:

1. History of coronectomy on one or both mandibular 3M within the specified timeframe.
2. Routine follow-ups at specified time intervals after coronectomy, that is, within first month, after 6 months and at a 1-year interval or later.
3. Radiographic evaluation was performed only on the participants who had a preoperative and post operative OPG (at intervals of either 6 months and 1 year or both).
4. Only patients who agreed to participate in the study and signed the written consent form were included in the study.

OPG was the standard radiographic modality for evaluation of 3Ms at the Department of Oral and Maxillofacial Radiology, and all radiographic images were provided with a written interpretation by the radiologists.

### Study variables & data collection

Demographic variables such as age and gender were recorded. Indications for coronectomy procedure, tooth number (FDI), post-operative infections, nerve damage, and use of antibiotics (pre- and post-operative) were recorded from EPJ. Radiographic findings such as tooth position and morphology, root shape, and pre- and post-operative radiographical changes were analysed from the OPG. Visible radiographic root migration at 6 months intervals and 1-year follow-ups or later were also analysed from the OPG. Data were collected and organised into an Excel spreadsheet.

### Surgical procedure

Preoperative mouth rinsing with 0.2% chlorhexidine (Corsodyl 2 mg/ml, GlaxoSmithKline AS, Oslo, Norway) was performed for 1 min before the surgery. Patients were routinely prescribed 1 g Paracetamol and 600 mg ibuprofen if their medical history allowed the use of these medicines without any interactions or contraindications. Surgery was exclusively performed under local anaesthesia (Xylocaine Dental Adrenaline, Dentsply Ltd., Surrey, England). As a standard coronectomy procedure, the mucoperiosteal flap was raised with a distal releasing incision; bone was removed buccally to gain access to the coronal part; the crown was sliced at or below the cementoenamel junction leaving no enamel rests. The root system was reduced to at least 2–3 mm below the marginal bone level, and any granulation tissue was removed. If mobility of the root fragment was observed intraoperatively, the procedure was considered unsuccessful and surgical extraction of the root complex was performed instead. Primary closure of the wound was achieved with the help of resorbable sutures (Vicryl 4-0, Ethicon). Patients were scheduled for post-operative follow-up 7–10 days after the procedure, and clinical and radiographic findings were noted in the EPJ.

### Sample size

**Figure 1:**
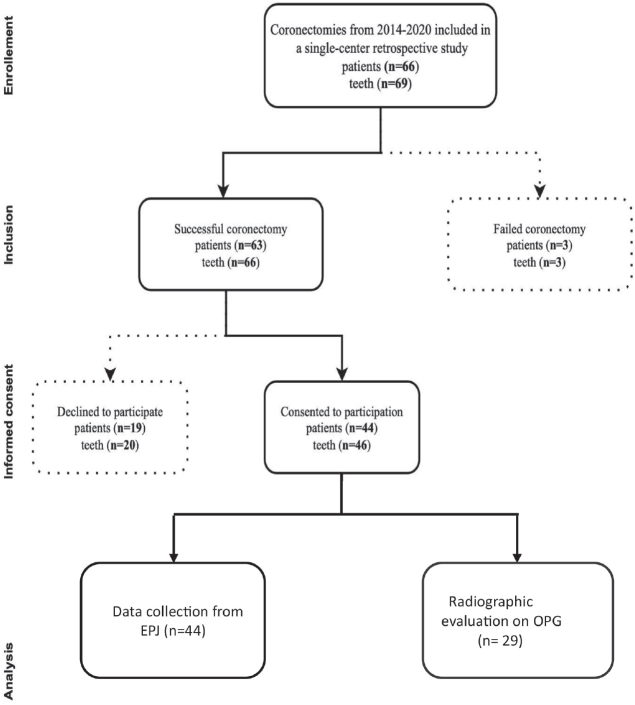
Flowchart illustrating the sample size collection.

The study population consisted of all patients who underwent coronectomy between 2014 and 2020 at the Department of Oral and Maxillofacial Surgery, IKO, UiB. Coronectomy was performed on 63 patients, and consent forms were sent to all the individuals. A total of 44 patients (69.8%) signed the written consent forms, and were included in the study. Demographic variables and clinical data about infections and complications were collected from the EPJ of the 44 patients. However, only 29 of these participants had both pre- and post-operative OPG and were included for radiographic analysis (Figure 1). The rest of the participants (*n* = 15) had either 3D radiographs or did not have a follow up OPG. Figure 1 shows the sample size available for the study.

### Radiographical analysis

Pre- and post-operative OPG of the 29 patients were retrieved for radiographic analysis. Two observers from the final year dental student class underwent a training session to evaluate the OPG under the supervision of a team consisting of an oral and maxillofacial surgeon and a radiologist. The two students then underwent a calibration session to standardise a meticulous analysis pattern. A methodical approach was followed for analysing the OPG, including systematic evaluation of all the panoramic radiographs, inspecting the tooth position in the jawbone, tooth direction and impaction, anatomic location of the nerve canal in relation to roots, and taking into consideration the post-operative changes, that is, osseous changes and migration of roots in relation to the neurosensory bundle. The two observers analysed the OPGs from 29 patients in two sessions, and mean results from both observers were recorded and compared. Table 1 shows the mean agreement between the two observers. As mentioned earlier, uncertain results were recorded as ‘no’. For these data, the two observers evaluated each of the 3 questions below twice. Due to the repeated measurement structure of the data, we set up a random effect logistic regression with question within observer as a random factor. Hence, we measure the agreement for each of the questions. The random effects model will also account for missingness in the data. Based on the random effect logistic regression we calculated the agreement as Intra class correlation (ICC). The overall ICC was found to be 0.81 (95% confidence interval [CI]: 0.68, 0.89).

**Table 1:** Mean percentage of agreement between the two observers.

Agreed (%)	*N*	%
100	75	66.37
75	27	23.89
50	11	9.73
	113	100.00

N = number of OPG observations.

The following parameters were used to assess whether there was visible root migration [16].

1. Presence of bone superior to the root complex (yes/no).
2. Superimposition between the root complex and the mandibular canal (yes/no).
3. Visible migration between root apex and the inferior alveolar canal (yes/no).

The following considerations were agreed upon in the calibration sessions in consultation with oral and maxillofacial radiologist to ensure dependable inter-rater reliability.

1. If the root complex has two or more roots, the most visible and identifiable root apex was chosen for the registration of findings.
2. The answer was registered as ‘no’ if there was uncertainty about the visible migration of roots.

Analysis was conducted twice per observer in two blinded sessions with an interval of 2 weeks in between sessions to avoid recall bias. Analysis was performed at the Department of Oral and Maxillofacial Radiology, where optimum light conditions and monitoring screens were provided in the isolated rooms for the best possible radiographic analysis. The magnification of ×1.0 was adjusted as standard on OPG. Radiographs were analysed using the Digora database, the default x-ray software at IKO. A minimum of two post-operative panoramic images were analysed for each patient. In the case of multiple OPG, images closest to 6 months (minimum 5 months after the procedure) and 1-year post-operative intervals were chosen.

### Statistical analysis

Microsoft Excel 365 version 2305 was used for data entry, and analyses was performed using IBM SPSS Statistics for Windows, version 28.0. Armonk, NY: IBM Corp. Percentage was calculated for categorical variables, while mean and standard deviation (SD) were calculated for continuous variables. Pearson chi-square test or Fishers exact test was calculated to analyse any statistical difference between gender, age, root anatomy, and tooth position. For all tests, the significance level was set to 5% (α = 0.05).

## Results

There were 63 coronectomy procedures performed at IKO during the period 2014–2020. A total of 44 patients (69.8%) were included in this study (24 females and 20 males; mean age 28.5 years; range: 18–55 years), as shown in Table 2. In 24 cases, the right-side 3M was treated while in 22 cases, the left side was treated. Two patients had the procedure performed bilaterally.

**Table 2:** Patient’s demographics and baseline characteristics of 3Ms.

Characteristics	
**Age** * N*, (Mean, SD)	44 (28.6, 7.99)
**Gender *N*, (%)**	
Female	24 (54.5)
Male	20 (45.4)
**Tooth number^1^ *N*, (%)**	
38	22 (47.8)
48	24 (52.1)
**Pattern of impaction *N*, (%)**	
Vertical	18 (39.1)
Mesioangular	19 (41.3)
Distoangular	9 (19.6)
**Root anatomy *N*, (%)**	
Fused	16 (34.7)
Divergent	30 (65.2)

The follow-up period would normally be after 1 week, 6 and 12 months, but it was inconsistent in our study because of the predominantly student patient population, with a relatively high loss to follow-up ratio. The longest follow-up was recorded at 1,028 days. There were 103 post-operative follow-ups with an average of 2.24 visits per patient (SD = 1.25). A total of 86.9% had one or more post-operative visits within the first month of the procedure.

Patient demographic data and the anatomical variation of 3M teeth are shown in Table 2. Most of the 3M had either a mesioangular (41.3%, *n* = 19) or vertical position (39.1%, *n* = 18). The remaining 19.6% (*n* = 9) had a distoangular position. In most cases, the root form was divergent (65.2%, *n* = 30), while the remaining had a fused root anatomy (34.8%, *n* = 16).

### Root migration

Visible root migration was observed in a total of 72.4% (*n* = 21) of 29 teeth. This incidence of root migration was found to be equally distributed among both females and males (Table 3). The average age of the patients with migration was 26.7 ± 7.68 years (SD = 7.68, range: 18–55). Root migration was observed considerably more often in the younger group (25 years old or younger) than in the older age group (*p* = 0.044).

**Table 3:** Findings based on EPJ’s and OPG’s.

Variables	Root Yes (%)	Migration No (%)	*p*
**Gender *N*, (%)**			
Male	10 (34.5)	3 (10.34)	0.69^a^
Female	11 (37.9)	5 (17.2)	
**Age *N*, (%)**			
≤25	12 (41.4)	1 (3.45)	0.044^a^
≥26	9 (31.0)	7 (24.13)	
**Root anatomy *N*, (%)**			
Fused root complex	8 (27.5)	3 (10.3)	1.00^a^
Divergent	13 (44.8)	5 (17.2)	
**Tooth position *N*, (%)**			
Mesioangular	9 (31.0)	3 (10.34)	0.83^b^
Vertical	8 (27.5)	4 (13.8)	
Distal	4 (13.8)	1 (3.45)	

a Fisher’s exact test.

b Pearson chi square test.

**Figure 2:**
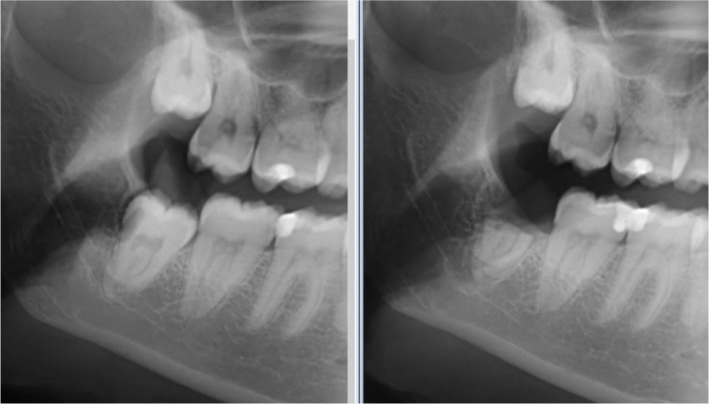
Preoperative and post-operative image after coronectomy (right). The image on the right side shows the superior movement of the root complex away from the nerve in a superior direction.

Root migration was assessed in relation to root anatomy, that is, fused root complex as a cluster or divergent roots. Root migration was observed in 44.8% of teeth with divergent roots and 27.5% of teeth with fused root complex. Root anatomy does not seem to affect root migration. In addition, tooth position was analysed to determine if it affects root migration. It was observed that the direction of root migration depends on the tooth’s position in the socket, and follows the same course during migration. Radiological analysis showed that nine roots migrated in a mesial direction, four in a distal direction, and eight in a vertical direction corresponding to tooth’s impaction, as mentioned in Table 3. The presence of bone superior to the root complex was observed in 62.1% (*n* = 18) of cases. In 42.9% (*n* = 9) of the cases with root migration, one could observe that the root complex migrated in the coronal direction away from the superimposition of the inferior alveolar canal. This is shown in an example in Figure 2.

### Use of antibiotics

A total of 8 patients (17.4%) out of 44 were prescribed with post-operative antibiotics. Of these eight patients, six patients (75.0%) were prescribed antibiotics on prophylactic grounds, that is, phenoxy methyl penicillin 1 g four times a day for 5–7 days course while two patients received antibiotics because of post-operative infection.

### Post-operative complication

In this study, post-operative complications were divided into three categories: (1) nerve damage or any alteration to neurosensory innervation, (2) post-operative infection, and (3) need for removal of the root complex in a second operation, for example, because of infection or root exposure.

There was no reported case of transient or permanent nerve injury out of the 46 cases. Post-operative infection was recorded in 8.7% (*n* = 4) of the patients. Two of these cases resolved with systematic antibiotics, and the healing process continued uninterrupted. Remaining roots were removed in two cases, that is, in 4.34% of cases because of unresolved post-operative infection. No roots were removed in a secondary operation because of root exposure in this study.

## Discussion

**Figure 3:**
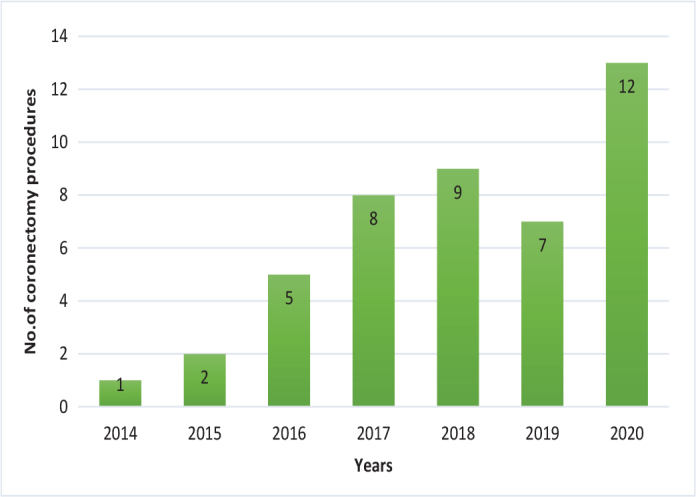
Number of coronectomy procedures performed at IKO over the years.

Coronectomy is a relatively rare procedure despite its introduction in 1984. However, the number of cases performed has gradually increased over the years. Data from this study show 63 procedures were performed over a period of 7 years while careful estimates from the same department reveal that almost 1,200 surgical extractions are performed per year; thus, there were approximately 133 surgical extractions per coronectomy procedure (Figure 3). This study was designed to evaluate the outcomes of coronectomy and to identify any factors that influence root migration, and to document the occurrence of infection or other complications after surgery.

OPG is used as a standard radiographic modality for 3M evaluation at our institution. This is because the projection is standardised each time a new image is taken, and the resulting images are comparable. Moreover, the radiation dose in conventional OPG is considerably lower than cone beam computerised tomography (CBCT), and in keeping with the ALARA principle that radiation exposure be kept ‘as low as reasonably achievable’, it is desirable to achieve the diagnostic goals by keeping the radiation exposure at minimum [20]. Guidelines regarding CBCT for 3Ms from the European Academy of DentoMaxilloFacial Radiology (EADMFR) (2019) recommend ‘great restraint’ in the use of CBCT and suggest that CBCT should be exclusively used in exceptional cases to answer a specific clinical question that cannot be answered by conventional OPG [21].

Although, CBCT is the more accurate modality for measuring the root migration distance, OPGs were used instead to measure the visible root migration as was done in a previous study by Pedersen et al. [11].

**Figure 4:**
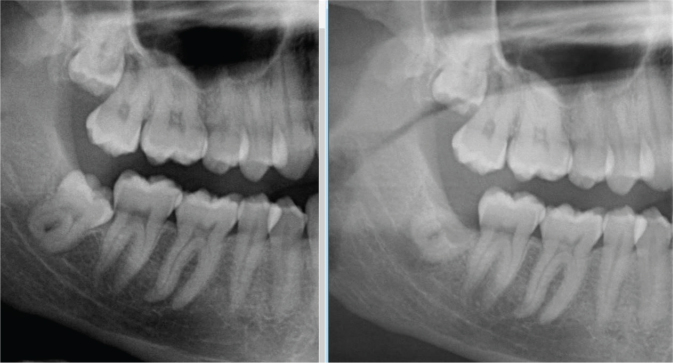
Visible root migration after coronectomy procedure.

Long-term follow-up studies on coronectomy are limited, but the available studies show that maximum root migration in terms of distance occurs within the first year, and migration gradually slows down afterward and stabilises, and there is almost little to no movement after 2 years [3, 22–24]. Figure 4 shows the movement of remaining roots in a cranial direction after a coronectomy procedure.

One of the drawbacks of coronectomy is the need for repeated follow-up. There is no consensus about radiological follow-up after coronectomy [16]. Suggested follow-up varies, and an interval of 3 months, 6 months, 1 year, and even long-term follow-up (3–5 years) are advised [11].

This study’s findings are consistent with previous studies and emphasise that coronectomy must be considered as a viable treatment option when there is a potential risk of nerve injury with traditional surgical treatment [14]. There is convincing literature and clinical studies that show coronectomy is a safe procedure for lower 3M in proximity to IAN [11, 14, 25]. Future research should focus on the correlation between root migration and different demographic factors that could shed more information on the corroborating factors.

### Visual assessment of remaining roots

The parameter of superimposition between the root complex and mandibular canal was used by Pedersen et al. [16]. According to earlier studies, the authors observed visible signs of bone formation coronal to the root complex. Other studies have registered bone formation coronal to root complex in up to 99.0 and 97.5% of cases, respectively [11, 22].

Root migration in our study was observed in 72.4% (*n* = 21) of 29 teeth; this result is in accordance with an earlier systematic review where the root migration rate was registered between 30 and 85% [14]. In another meta-analysis, the root migration rate was observed between 30 and 75% [15]. In a recent study conducted by Pedersen et al., root migration was reported in 83.8% of cases after 1 year and no noticeable migration thereafter, with a mean observation period of 5.7 years [11].

### Age and root migration

Earlier clinical studies have shown that there is a correlation between age and migration distance of the remnant roots. This finding agrees with the results from our study where people in the younger group showed more root migration compared to the older group. Leung and Cheung [26] and Pedersen [16] have come to the same conclusion that there is an increased tendency for root migration among younger patients when compared to older patients. Yan et al. found that root migration was significantly lower in patients older than 24 years than in younger patients [27]. In a prospective study from the University of Oslo, where neurosensory disturbances were analysed after surgical extraction of 1,220 3M, it was concluded that the risk of developing permanent nerve damage was considerably lower in people younger than 30 years of age [28]. Osborn et al. have also reported a higher risk of neurosensory disturbances (6.5%) in patients older than 25 years [29].

### Tooth impaction and root migration

Due to the small sample size, the association between visible root migration and the type of impaction of 3M could not be ascertained. Of the migrated remnant roots included in the study, 43% had a vertical position, 38% had a mesioangular position, and 19% had a distoangular position. However, no association was observed between tooth impaction and root migration.

### Antibiotics

The antibiotic usage in this study was either on prophylactic grounds or for the treatment of post-operative infections. There is no standard protocol for the usage of antibiotics in coronectomy. However, studies have shown a reduced infection rate with the use of antibiotics [30]. Some practitioners did prescribe it as a prophylactic treatment [19, 31], while other authors used it purely for therapeutic reasons [3, 23, 32].

### Post-operative complications

In the present study, post-operative infections were observed in 8.7% of patients. A meta-analysis shows that post-operative infection after coronectomy lies between 0 and 0.81% [15]. The highest reported infection rate associated with coronectomy procedures was reported at 10.98% [33]. Several studies suggest that the risk for a post-operative infection after coronectomy is similar to that of total removal, and there is no significant difference in the use of prophylactic antibiotics [14, 26]. These findings are in accordance with the current study’s findings and do not support the use of prophylactic antibiotics with coronectomy.

### Removal of remaining root

In the present study, only three remnant roots were removed in a total of two patients (one patient with coronectomy performed on each side) out of 46 coronectomy procedures performed.

Removal of remaining root was done in less than 5% of patients in this study, which is in accordance with the literature. Cosola et al. did a 4-year follow-up study and concluded that the removal of root was necessary in 5% of the cases [18]. There were no reported cases of root removal because of root migration in our study. However, other researchers have reported a range of 0 to 5.1%, respectively for removal of remaining root [25, 34].

### Nerve damage

There were no reported cases of transient or permanent nerve damage in this study. Similar results were seen in a case report by Cosola et al. [18]. Pedersen et al. reported two cases of hypoesthesia out of 200 cases over a follow-up of 5 years [11]. No other cases of permanent nerve damage were found in other studies [30]. Reported transient nerve damage in successful coronectomies ranges from 0 to a maximum of 2.2% [25, 35, 36]. However, reported cases of temporary nerve damage in failed coronectomy are considerably higher and range from 6.25 to 8.0%, respectively [3, 25]. It was concluded that successful coronectomy decreases the risk of mechanical trauma to the nerve [11].

### Limitations of this study

This study was done at a university dental clinic where a majority of the patients were young students, which led to a large loss to follow-up. Furthermore, there is no consensus or a standardised follow-up protocol after coronectomy. Acquiring descriptive information on root migration from two-dimensional (2D) images is another limitation in this study, because 2D images tend to overlap surrounding anatomical structures.

## Conclusion

This study concludes that coronectomy is a safe procedure with a low risk of complications. This study also highlights that age is an important factor affecting root migration, and coronectomy in people younger than 25 years should only be considered on strong indications since the risk of permanent nerve injury in this age group is low. There are no established guidelines regarding the prophylactic use of antibiotics, and the findings of this study suggests that antibiotics be used only on therapeutic grounds rather than prophylactic. Furthermore, this study indicates the need for a standardised post operative follow-up after coronectomy. Based on the cited literature, it is suggested that post-operative follow-ups should be conducted at 1 week interval after the procedure with a baseline radiograph and at 1 year interval to analyse the root migration. Further, patients can be followed up by a general dental practitioner at regular checkups.
