# Correction of author list for article: *A retrospective study analysing outcomes of the coronectomy procedure at a university dental teaching clinic in Norway*

**DOI:** 10.2340/aos.v84.44706

**Published:** 2025-09-03

The corresponding author of article: Sharif U, Lie SA, Gjerde CG. *A retrospective study analysing outcomes of the coronectomy procedure at a university dental teaching clinic in Norway*. Acta Odontol Scand. 2025 May 26;84:284-291. doi: 10.2340/aos.v84.43759. PMID: 40418032; PMCID: PMC12138385, requested a correction in the author list. During the manuscript preparation, they were not asked to participate in the writing phase due to their limited availability at the time. Consequently, their names were not included among the listed authors in the published version, though their contributions were acknowledged in the Acknowledgments section. The correction was made in agreement with all co-authors.

The new author list is: Umar Sharif, Johannes Finne, Nourhanim Fadul, Stein Atle Lie and Cecilie Gjerde.

